# Crosstalk between cGAS-STING pathway and autophagy in cancer immunity

**DOI:** 10.3389/fimmu.2023.1139595

**Published:** 2023-03-01

**Authors:** Qijun Lu, Yukun Chen, Jianwen Li, Feng Zhu, Zhan Zheng

**Affiliations:** ^1^ Department of Oncology, Longhua Hospital, Shanghai University of Traditional Chinese Medicine, Shanghai, China; ^2^ Cancer Institute, Longhua Hospital, Shanghai University of Traditional Chinese Medicine, Shanghai, China; ^3^ Department of Laboratory Medicine, Huadong Hospital, Fudan University, Shanghai, China

**Keywords:** antitumor, autophagy, cancer, cGAS-STING, immunity

## Abstract

The cyclic GMP-AMP synthase-stimulator of interferon genes (cGAS-STING) pathway is critical in cancer immunity. Autophagy is a highly conserved process that is responsible for the degradation of cytoplasmic material and is involved in both innate and adaptive immunity. Recently, cGAS-STING and autophagy have been shown to be interconnected, which may influence the progression of cancer. Although cGAS-STING and autophagy have been shown to be interrelated in innate immunity, little has been reported about cancer immunity. As cancer immunity is key to treating tumors, it is essential to summarize the relationship and interactions between the two. Based on this, we systematically sorted out the recent findings of cGAS-STING and autophagy in cancer immunity and explored the interactions between cGAS-STING and autophagy, although these interactions have not been extensively studied. Lastly, we provide an outlook on how cGAS-STING and autophagy can be combined, with the hope that our research can help people better understand their potential roles in cancer immunity and bring light to the treatment of cancer.

## 1 Introduction

Cancer is one of the world’s most serious threats to human health, with high morbidity and mortality rates, and according to the latest global data, 9.96 million patients will die from cancer in 2020 (1). Cancer is a genetic abnormal disease triggered by a long-term combination of multiple factors. When the human body is affected by chemical, physical, viral, and other carcinogenic substances in the environment or due to its own genetic, endocrine, gender, age, and other factors, a series of abnormal genetic changes will occur to form malignant tumors (2). Tumor cell growth is initiated by mutations that activate oncogenic drivers. This process is combined with the genetic or non-genetic activation or inactivation of genes that promote or inhibit tumor proliferation (3). In many cancers, oncogenesis is accompanied by the accumulation of mutations, which can provide a selective advantage to cancer cell populations by increasing the degree of genetic diversity and accelerating their evolutionary adaptation (4, 5). However, this diversity comes at a cost: the more the cancer cell differs from normal cells, the more likely it is to be recognized as a foreign agent by the immune system.

Current clinical treatment of malignancies is still dominated by radiotherapy, chemotherapy and surgery, but the 5-year survival rate of patients is still very low (6). Along with the advancement of human understanding of tumor immunity, immunotherapy has become increasingly sophisticated and offers new hope for cancer treatment (7–10). Immune checkpoint inhibitors, such as therapeutic monoclonal antibodies targeting the programmed cell death protein 1/programmed cell death ligand 1 (PD-1/PD-L1) pathway, have been approved as monotherapy or combination therapy for oncology treatment (11). One of the main targets of immune checkpoint inhibitors is the release of effector T cells. The positive correlation between T-cell infiltration in the tumor stroma and prognosis, as well as the clinical success of chimeric antigen receptor (CAR) T-cell infusion in certain hematologic malignancies, suggest a critical role for T cells in tumor immunity (12). These clinical successes have led to a T-cell-centric view of tumor immunity. There is a strong link between cancer and the immune system (13). Adaptive immunity, as well as innate immunity, make up the immune system. However, the effector function of T cells is not autonomous (14). The immune system promotes or suppresses tumor growth by recognizing and killing cancer cells. the initiation and maintenance of T cell responses and the development of durable protective memory T cells are dependent on the innate immune response (15). Innate immunity involves various types of myeloid cells, including dendritic cells (DCs), monocytes, macrophages, polymorphonuclear cells, mast cells, and innate lymphocytes (ILCs), such as natural killer (NK) cells (14). Innate immunity is the host’s first and fastest line of defense against invading pathogens. Different pattern recognition receptors (PRRs) are used to activate the innate immune response when host cells recognize conserved pathogen patterns known as pathogen-associated molecular patterns (PAMPs) and danger-associated molecular patterns (DAMPs) (16). In eukaryotic cells, DNA is usually present in the nucleus and mitochondria. The DNA present in the cytoplasm is usually due to microbial infection or DNA damage. Thus, cytoplasmic DNA is a red flag that triggers a strong innate immune response (17). Recognition of cytoplasmic DNA is an important host defense mechanism. Cyclic GMP-AMP synthase (cGAS) is thought to be a key sensor mediating cytoplasmic DNA recognition.

**Figure 1:**
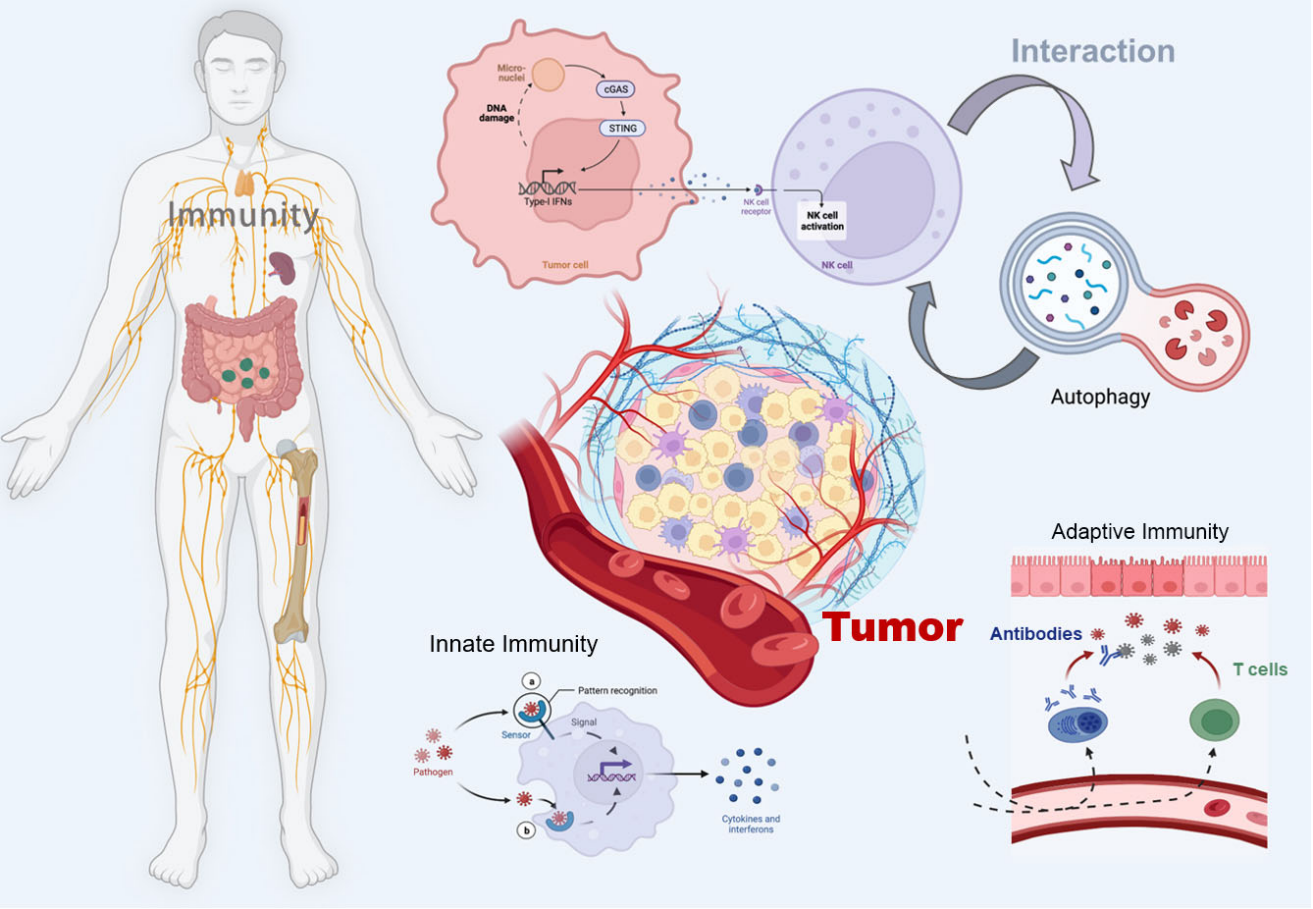
Schematic illustration of the crosstalk between the cGAS-STING pathway and autophagy in cancer immunity. The figure was created with BioRender (https://biorender.com/).

The STING pathway has emerged as a promising drug target for the treatment of cancer (18). By triggering the cGAS-STING pathway, the innate immune system can be activated, promoting acquired immunity to fight cancer and thus improving survival (19). In addition, autophagy, a tightly regulated mechanism of cellular self-degradation, is essential for maintaining intracellular homeostasis under stressful conditions (20). Autophagy is extensively involved in the survival, development, and maturation of immune cells (21). It can contribute to the initiation or inhibition of tumor growth by regulating the development of innate and adaptive immunity (22). cGAS-STING pathway can trigger autophagy in several ways, and autophagy can also regulate the cGAS-STING pathway (23). Therefore, in this review, we systematically discuss the interaction between the cGAS-STING signaling pathway and autophagy in cancer immunity, hoping to provide a direction for exploring new cancer immune mechanisms and therapeutic approaches (**Figure 1**).

## 2 Overview of the cGAS-STING pathway

The immune system recognizes different pathogens to protect the body and maintain homeostasis. Innate immunity functions as the first line of defense against pathogenic microorganisms and as a basis for adaptive immune responses. The host cells become aware of a pathogen invasion through pattern recognition receptors, which will initiate a series of signaling events. Many pattern recognition receptors exist, such as Toll-like receptors, Nod-like receptors, and Scavenger receptors. A recently discovered pathogen recognition receptor, cyclic guanosine monophosphate-adenosine monophosphate synthase (cGAS), can activate any sequence of double-stranded DNA (dsDNA) (24) and participate in various cellular processes, including proliferation, apoptosis, differentiation, and invasion of cancer cells.

**Figure 2:**
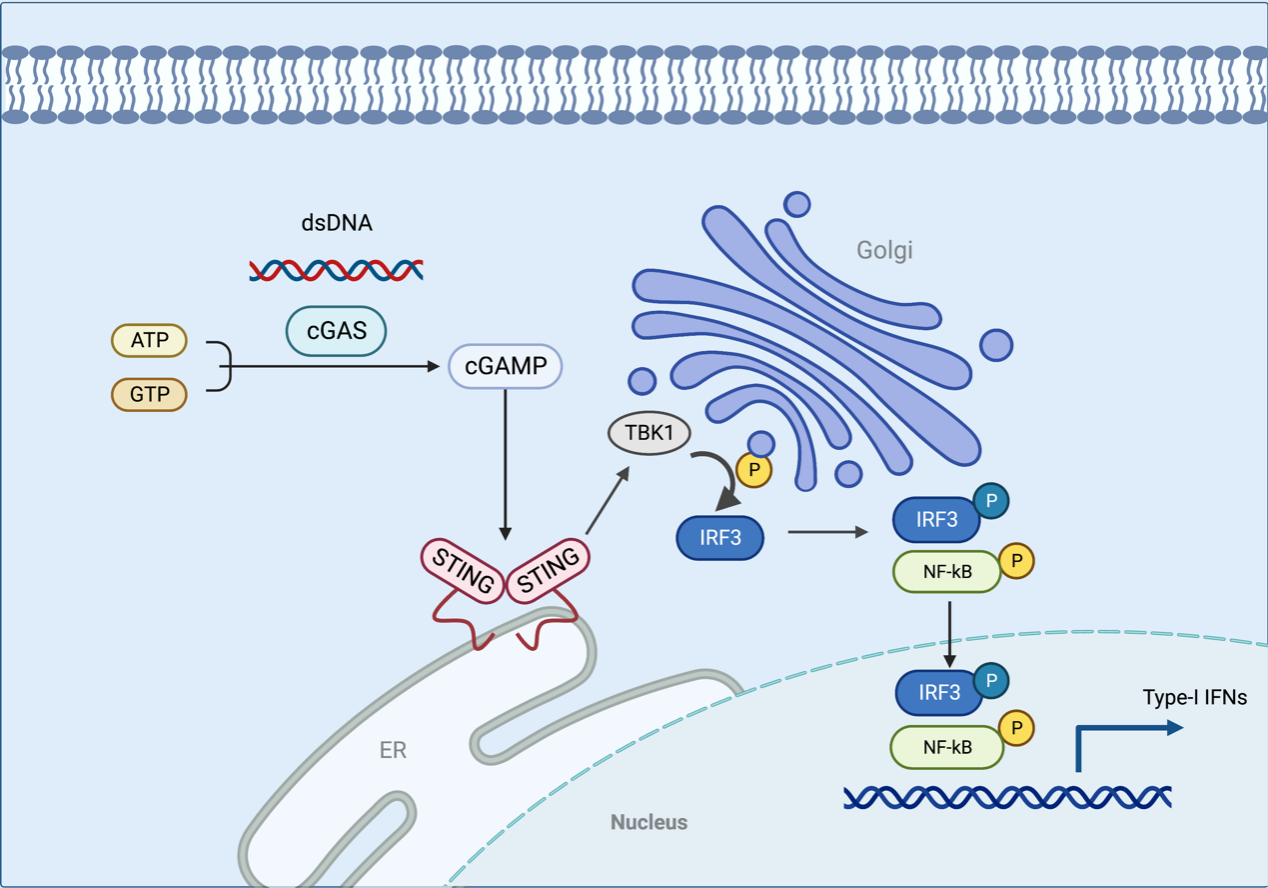
In this model, the cGAS interacts with the dsDNA via liquid-liquid phase separation, which activates the cGAS. STING is activated in the ER when cGAMP is generated in response to the concentration of the reactants. As STING is transferred to the Golgi apparatus, TBK1 is recruited to activate IRF3. When IRF3 is activated, it enters the nucleus and functions with NF-κB to produce type I IFN. The figure was created with BioRender (https://biorender.com/).

STING is a receptor protein located on the endoplasmic reticulum (ER) that is critical for the response pathway in innate immunity. It is usually observed in the resting state as a dimer. By liquid-liquid phase separation, cGAS and dsDNA interact to form micrometer-sized drops that activate cGAS. As the reactants concentrate, these lipid droplets generate cyclic guanosine monophosphate-adenosine monophosphate (cGAMP) which can be catalyzed from ATP and GTP (25, 26). STING is activated by cGAMP in the ER and becomes a tetramer by oligomerization (27). Sulfated glycosaminoglycans induce STING to translocate into the Golgi apparatus and perinuclear endosomes from the ER (28), during which STING is palmitoylated in the Golgi apparatus and caused to activate IRF3 by recruiting TBK1 kinase, which undergoes transautophosphorylation, thus enhancing affinity for interferon (IFN) regulatory factors. When IRF3 is activated, it enters the nucleus, which works synergistically with NF-κB to promote the transcription of type I IFN genes and related immunomodulatory factors (29–31). STING is rapidly degraded by sorting into lysosomes after signaling (32). In addition, STING can mediate the activation of the NF-κB pathway downstream of DNA damage signaling independently of cGAS, and the E3 ubiquitin ligase TRAF6, P53, DNA damage kinase ataxia telangiectasia mutated, enzyme poly(ADP-ribose) polymerase 1, and interferon-γ-inducible factor 16 combine to form a distinct STING signaling complex that induces TRAF6-dependent NF-κB activation (33–37). However, the exact mechanism of STING-dependent NF-κB pathway activation remains unknown (**Figure 2**).

## 3 cGAS-STING pathway in cancer

Many tissues have been found to express STING, such as the heart, spleen, lung, ovary, and various antigen-presenting cells (APCs). However, it was less expressed in tissues such as the brain, liver, kidney, small intestine, and colon. According to The Cancer Genome Atlas dataset, cGAS and STING gene expression was detected in all types of cancer, but the expression varied according to the stage and type of cancer (38). STING expression is significantly increased in murine pancreatic cancer models and human pancreatic tumors, as well as tongue squamous cell carcinoma, while down-regulated in malignant melanoma (39, 40). In addition, patients with lung adenocarcinoma had lower cGAS expression and longer survival (41). Based on the evidence presented above, cGAS-STING is inextricably linked to cancer.

In further studies, cGAS-STING was found to have a tumor-suppressive effect. By regulating the initiation of intestinal inflammation, STING may hinder the progression of colon cancer, and it may also regulate various signaling pathways such as signal transducer and activator of transcription-3 and NF-κB (42). However, tumors can develop when the cGAS-STING pathway is overactivated. By activating STING, the carcinogen 7,12-dimethyl-Benz[a]anthracene can cause DNA breaks in mice, resulting in skin tumors (43). In the same way, STING activation is associated with Lewis lung cancer growth (44).

Furthermore, the cGAS-STING pathway is involved in cancer metastasis. Cancer cells can transfer cGAMP to astrocytes *via* the cancer-astrocyte gap junction channel, which activates STING in astrocytes and subsequently produces inflammatory cytokines such as IFN-α and TNF-α, which in turn activate signal transducers and activator of transcription 1 (STAT1) and NF-κB signaling pathways in the cancer cell, leading to brain metastasis (45). In metastatic breast cancer, cGAS-STING signaling can activate atypical NF-κB pathways, which can promote metastasis due to epithelial-mesenchymal transition (EMT) (46). Meanwhile, the elimination of STING can inhibit breast cancer metastasis by reducing the expression of the EMT gene (46).

## 4 cGAS-STING pathway in cancer immunity

cGAS-STING participates in the remodeling of the tumor microenvironment (TME) (47), which induces the production of antitumor cytokines such as interleukin 10 and invariant surface glycoprotein (ISG) that inhibit tumor growth (48). Macrophages serve as powerful APCs by engulfing foreign pathogens and priming host defenses (49). The cGAS-STING pathway could significantly regulate macrophage polarization, which is considered an essential part of innate immunity and may be adopted as a target for immunotherapy-related diseases. Administration of liposome-derived cGAMP nanoparticles (cGAMP-NP) to tumor cells can activate STING in macrophages, repolarize M2-type macrophages into M1-type macrophages, improve MHC-like molecules or costimulatory molecules, and then induce differentiation of CD4^+^ and CD8^+^ T cells, thus producing a potent antitumor response (50).

In tumor cells, activating the cGAS-STING pass-through may inhibit the development of early tumors by upregulating type I IFN and other inflammatory genes. TME contains multiple proangiogenic factors that stimulate the formation of new blood vessels during tumor angiogenesis (51). Endothelial STING controls T-cell transendothelial migration in association with IFN-I (52). Activating STING increases the immune response to the TME and normalizes the tumor vasculature. In addition, the cGAS-STING pathway affects CD8^+^ T cell-mediated antitumor immunity by type I IFN. Downregulation of the cGAS-STING pathway leads to a reduction in tumor-infiltrating CD3^+^ CD8^+^ T cells by inhibiting type I IFN downstream genes, including chemokine ligands 9 and 10 (53).

In the immune system, DCs also play an essential antitumor immunity role. The STING protein in DCs amplifies signals from cytoplasmic DNA sensors, enhancing the adaptive immune system of the tumor. After being absorbed by tumor-infiltrating DCs, exosomal DNA activates STING signaling (54). DCs respond to NARK signaling by phagocytosing dead/damaged tumor cells, transferring exosomes, and forming cGAMP gap junctions. After injecting type I IFN, DCs drain lymph nodes and trigger tumor specific CD8^+^ T cells to migrate to the tumor. Finally, these CD8^+^ T cells proliferate in lymph nodes, killing the tumor cells (55). During TME, phagosomes degrade mtDNA from tumor cells, causing the production of type I IFN in the DC cytoplasm; inhibiting CD47 suppresses this degradation, enhancing adaptive immunity against tumors (56). If STING is deleted in DC, the ability to present antigens is abolished, and tumor infiltrating lymphocyte abundance is decreased (57). A similar effect was observed in colon tumors with MC38 after radiation exposure by mobilizing myeloid-derived suppressor cells (MDSCs) dependent on the host STING molecule (58).

In contrast, cGAS-STING signaling may promote tumor growth and metastasis. Chronic activation may induce an immunosuppressive TME (17). STING was associated with poor prognosis in a subset of patients with colorectal cancer (38), suggesting that STING may contribute to tumor growth and immune evasion. Recent research found that STING agonists activate cell stress in T cells and trigger cell death (59). Another study found that constitutive activation of STING impaired T lymphocyte proliferation, a process dependent on NF-kB and triggered by STING relocalization to the Golgi apparatus (60). These findings suggest that cGAS/STING, as an innate sensor, also has the potential to impair the adaptive immune system.

Immune responses to DNA in the TME are influenced by tumor antigenicity, which is underappreciated. Through the induction of indoleamine 2,3-dioxygenase (IDO), the cGAS-STING pathway promotes tumor progression with low antigenicity (44). However, it remains unclear how cGAS-STING signaling stimulates cells to express PD-L1, which is known to mediate immune evasion of cancer cells (61). Mutations in the liver kinase B1 (LKB1) cause primary resistance to immunotherapy in non-small cell lung cancer (NSCLC). When LKB1 is lost, STING is inhibited, and cytoplasmic dsDNA is not sensitive to detection. Cancers resistant to immune checkpoint blockade may benefit from reactivating the LKB1 or STING pathways (62). In tongue squamous cell carcinoma samples, STING expression increased with tumor progression, with STING protein activation seen in papillomavirus positive specimens. In contrast, STING gene silencing does not affect cell viability or apoptosis but promotes IL-10, IDO, and CCL22, thus enhancing immunosuppressive cytokines and regulatory T-cell infiltration, suggesting that STING regulates the TME and influencing tumor progression (63).

## 5 Overview of autophagy

Autophagy is a tightly regulated and stress-induced catabolic process that regulates cancer in eukaryotes (64). Macroautophagy, also known as canonical autophagy, can be divided into several stages including initiation, nucleation, or phagophore formation, elongation of the phagophore membrane to form the autophagosome, fusion of the autophagosome with the lysosomes, and degradation of the contents of the autophagosome (65). Macroautophagy was initially thought to be a massive degradation process activated by cellular starvation. Nevertheless, new findings suggest that autophagy also functions as a quality control mechanism for specific organelles and proteins (66). Through lysosomal or endosomal invagination, cytoplasmic cargo is engulfed during microautophagy (67).

**Figure 3:**
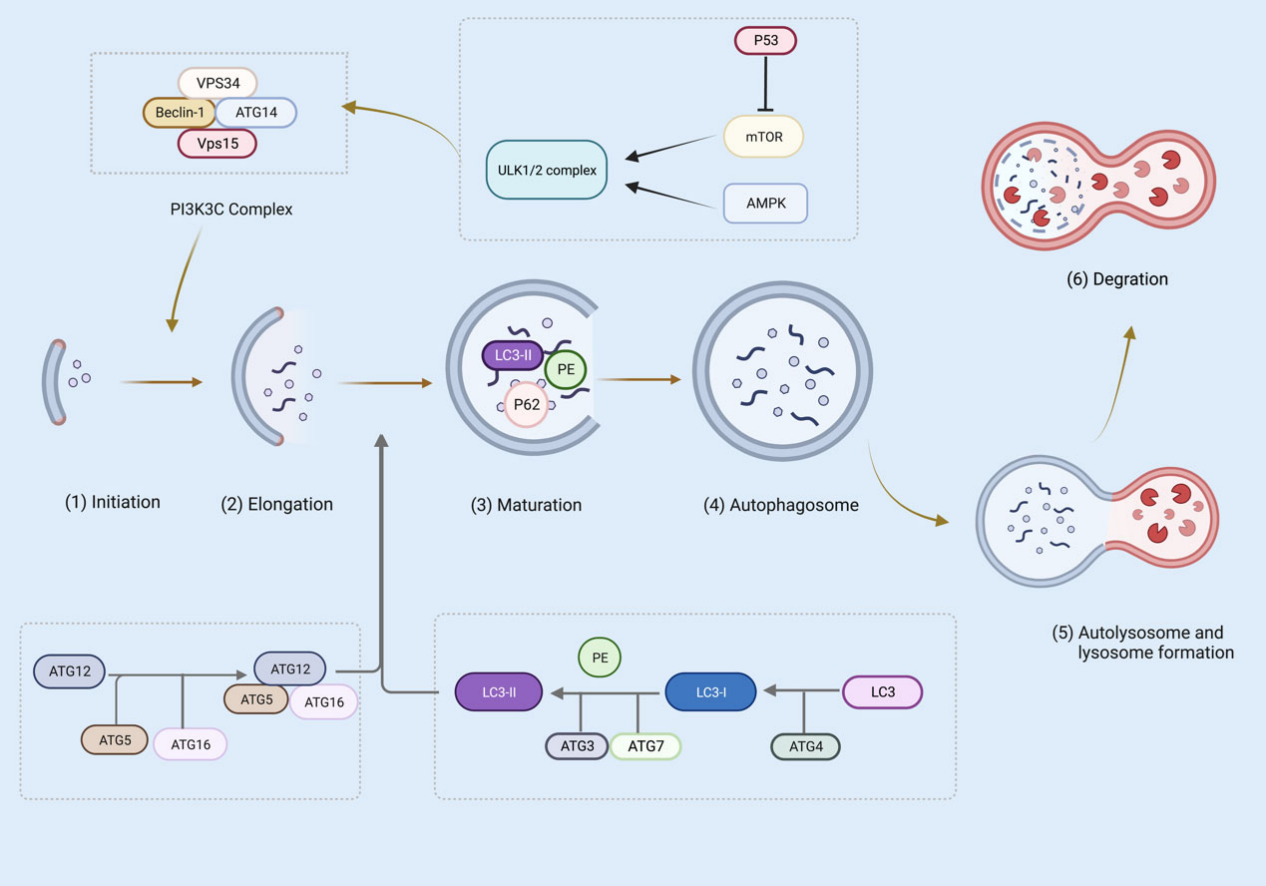
Several steps are involved in canonical autophagy: (1) initiation; (2) nucleation or phagosome extension; (3) maturation; (4) autophagosome formation; (5) autophagosome and lysosome formation; (6) degradation. The figure was created with BioRender (https://biorender.com/).

During canonical autophagy, signaling pathways such as mTOR and AMPK sense metabolic stress and thus activate the Unc-51-like kinase 1 (ULK1 and ULK2) complex (68–70). In the initiation phase, ULK1/2 activates VPS34 and the complexes of VPS34, VPS15, autophagy-related gene (ATG)14, Beclin-1, and P150 catalyze the production of phosphoinositol-3-phosphate, recruiting a further boost to the autophagic pathway (70–72). The phosphoinositide 3-kinase (PI3K) complex is responsible for the expansion and maturation of autophagic vesicles (73). Furthermore, ATG5-ATG12-ATG16 and the LC3 ubiquitin-like system contribute to the extension of autophagosome membranes (74). In particular, ATG5-ATG12 non-covalently binds to interact with ATG16 to form the ATG5-ATG12/ATG16 complex (75). ATG4, ATG7, and ATG3 cleave the precursors of LC3-like proteins, maturing and conjugating them with phosphatidylethanolamine (PE) to form LC3-II, which drives the elongation and closure of cell membranes and, ultimately, the formation of autophagosomes (76, 77). P62 *via* a LIR motif (LC3 interacting region) interacts with LC3. P62 has also an ubiquitin binding domain (UBD) and can bind to autophagy cargo (the ubiquitinated proteins). thus, P62 is an adaptor protein, linking LC3 to its cargo (78). At the maturity stage, LC3-II is digested and autophagosome forms that fuse with a lysosome, causing cell cargo degradation (76) (**Figure 3**).

Although canonical and non-canonical autophagy pathways share overlapping machinery, they differ in several important ways (79, 80). Non-canonical autophagy processes include microautophagy, chaperone-mediated autophagy (CMA), and LC3-associated phagocytosis (LAP) (81). Microautophagy occurs when lysosomes or vesicular endosomes directly engulf intracellular material for degradation (82). CMA is the process of binding intracytoplasmic proteins to molecular chaperones and transferring them to the lysosomal lumen, where lysosomal enzymes digest them (83). However, CMA is selective in removing proteins and is a soluble protein (84). During phagocytosis, pathogens engage extracellular receptors, such as Toll-like receptors, to initiate LAP, a non-canonical form of autophagy (85). Also, immune complexes and dying cells can trigger LAP (86). Furthermore, LAP is an important mediator in the response to immune tolerance, in addition to participating in the degradation of engulfed pathogens (87). With increased research on non-canonical autophagy, the concept of autophagy has been better understood and appreciated.

## 6 Autophagy in cancer

Cells need to adapt to environmental disturbances to maintain homeostasis in the body. In this process, autophagy serves as a recycling pathway that participates in the turnover of cellular components (88). Also, autophagy is crucial for cancer cell survival in conditions of nutrient and oxygen deprivation by degrading protein and lipid bulks for nutrient recycling (89–91). Tumor types and tumor models affect autophagy in cancer progression (89, 90). Defects in autophagy *in vivo* have been linked to an increased risk of tumor initiation (92). However, it is still unclear how autophagy-deficient tumors sustain their growth. Hepatocellular tumors are more likely to progress in autophagy-deficient livers when the group box1 is released from autophagy-deficient hepatocytes, which increases proliferation capacity (93). A lack of autophagy inhibits the killing of triple-negative breast cancer cells both *in vitro* and *in vivo* (94). Phosphorylation of Beclin1 controls autophagy and promotes or inhibits it (95). It is reported to have increased Beclin1 expression in cancer tissues in 110 patients with prostatic carcinoma, suggesting that autophagy could promote tumorigenesis (96).

Malignant tumors are closely linked to autophagy, especially the processes of recurrence, metastasis, and drug resistance (97). Cancer progression has been characterized by metastasis. Autophagy in metastasis is quite complex as a survival pathway and quality control mechanism. During the early stages of metastasis, autophagy serves primarily as a suppressor by restricting necrosis and mediating autophagic cell death (98). On the contrary, in the advanced stages of metastasis, autophagy as a dynamic degradation and recycling system can help to cope with intracellular and environmental stresses, such as hypoxia, nutrient shortage, or cancer therapy, thus favoring tumor progression. Moreover, Autophagy is upregulated in primary human glioblastoma, melanoma, esophageal cancer, and hepatocellular carcinoma upon progression to advanced metastatic disease, and autophagy markers in these cancers are associated with poor prognosis (99–101), indicating its importance throughout the metastatic cascade. Also, profilin 1 participates in cell proliferation and enhances autophagy-induced drug resistance by interacting with the Beclin1 complex in multiple myeloma (102).

## 7 Autophagy in cancer immunity

Autophagy influences tumorigenesis by modulating the formation of TME, and this microenvironment causes changes in autophagy signaling pathways in tumors, stroma, and innate immune cells (103). Depending on the characteristics of the tumor, autophagy can promote or suppress the immune response of the TME. Autophagy of these cells can enhance antitumor immune responses and immunotherapy. As a major innate effector component of early immunity, NKs play a crucial role. When NK cells develop, autophagy protects them by removing damaged mitochondria and reactive oxygen species (ROS) (104). As a result of its interaction with ATG7, phosphorylated Forkhead box O (FoxO) 1 induces autophagy in iNKs (104). NK cell maturation may be affected by autophagy when ATG7 and FoxO1 are disrupted in the cytosol of immature NK cells (105). CCL5 overexpression was associated with significantly improved long-term survival in patients with melanoma. Targeting autophagy in a CCL5-dependent manner improves NK cell infiltration and inhibits melanoma growth (106). Therefore, autophagy can act as an inhibitor of the expression of protumor and antitumor chemokines, thus differentially influencing tumor progression.

Autophagy is involved in the processing and presentation of major histocompatibility complex (MHC) molecular antigens and T cell-mediated immune responses, which contribute to tumorigenesis or antitumor immune responses. Pancreatic ductal adenocarcinoma (PDAC) cells are targeted for selective degradation by the autophagy cargo receptor neighbor of BRCA1, inhibiting antigen presentation and killing T cells. On the other hand, inhibition of autophagy restored MHC I surface levels, improved antigen presentation, enhanced antitumor T cell responses, and reduced tumor growth in syngeneic host mice (107). Unlike LAP and LANDO14, canonical autophagy is required for the degradation of MHC I. This suggests that tumor cells can evade immune surveillance through autophagy-mediated degradation of MHC I. A significant component of tumor-induced immunosuppression is MDSCs, which produce DCs, macrophages, and neutrophils. Autophagy deficiency enhances the immunogenic properties of tumor-derived tumor-infiltrating autophagy-deficient monocytic MDSCs through impaired lysosomal degradation of MHC II molecules (108). Consequently, inhibition of autophagy in MDSCs may be beneficial in the treatment of cancer; however, it remains challenging to target specific myeloid subpopulations in TME. The ubiquitination of MHC II in DC affects homeostasis, phenotype, cytokine production, and Ag proteolysis by DC, affecting Ag presentation and T-cell and Ab-mediated immunity (109). By interacting with antigen-processing pathways in DCs, autophagy can effectively modulate adaptive immunity. Through autophagy, organelles and apoptotic proteins are degraded, promoting T-cell development and survival. Furthermore, autophagy in DCs was shown to process tumors intracellularly for the presentation of MHC II to CD4^+^ T cells (110). Fusion of viral and tumor antigens into the LC3-II protein of ATG8, which is located in autophagosomal membranes, increases the presentation to CD4^+^ T cells (111). A CD4^+^ T helper cell activates CD8^+^ T cells primed by DCs. An effector CD8^+^ T cell lacking autophagy cannot establish long-term memory for effective antiviral immunity (112). Mice lacking the autophagy genes *Atg5*, *Atg14*, or *Atg16L1* suffer from synthetic tumor growth impairment (113). Also, *Atg5^-/-^* CD8^+^ T cells show enhanced glucose metabolism which results in altered histone methylation and higher transcription levels (113). In contrast, limiting glucose could inhibit the Atg5-dependent enhancement effector, therefore directly enhancing antitumor immunity *via* autophagy (113). In addition, DC activity can be inhibited by autophagy and antigen degradation. Through autophagy induction, the immune response is activated, inhibiting T cell activation after EMT and ROS (114, 115), affecting tumor killing. Inhibition of LAP in myeloid cells induces tumor-associated macrophages (TAMs) to develop a proinflammatory phenotype and increases phagocytosis of dying tumor cells, suggesting that LAP can increase immunity (116).

Furthermore, TME galectin-1 (Gal-1) improves tumor cell adhesion, invasiveness, angiogenesis, and immune evasion and contributes to tumor progression (117, 118). Through TLR2-activated secretory autophagy and MVB/Rab11/VAMP7-mediated vesicle trafficking, Hepatocellular carcinoma (HCC) cells stimulate TAMs to actively secrete Gal-1 (119). Autophagy-secreted Gal-1 promotes the growth of HCC in mice and is associated with a poor prognosis in patients with HCC (120). HCC cells can inhibit macrophage autophagy flux *in vitro* and stimulate the expression of PD-L1 (121). Another report shows that autophagy blockade drives PDAC to up-regulate and utilize the NRF2-induced alternative macrophagocytosis nutrient procurement pathway, which allows tumor cells to extract nutrients from extracellular sources and use them for energy production (122). As a result, combined autophagy and macropinocytosis inhibition may enhance cancer treatment.

## 8 Upstream pathway of cGAS-STING and autophagy

As part of autophagy induction, the core complex Beclin-1-PI3KC3 generates a PtdIns-3-P-rich membrane that recruits autophagy proteins and forms autophagosomes (123). Rubicon interacts with the Beclin-1-PI3KC3 core complex, negatively regulating autophagy and PI3KC3 lipid kinase activity (124). Rubicon competes with cGAS in conjunction with Beclin1. Binding of the central NTase domain of cGAS to the central CCD of Beclin 1 inhibits cGAMP synthesis and subsequent IFN production, as well as stimulates Rubicon release from the Beclin 1 complex, which induces autophagy by activating PI3KC3, clearing cytoplasmic dsDNA, inhibiting cGAS activation and sustained immune stimulation (125). In conclusion, cGAS and Beclin-1 interact to coordinate the IFN and autophagic pathways and thereby regulate the innate immune response.

cGAS contains five LC3-interacting regions (LIRs) that bind to LC3 and induce noncanonical autophagy (126). In a recent study, ATG7 and ATG14 were found to depend on the involvement of cGAS to contribute constitutively to nucleus clearance, suggesting that this pathway occurs through typical autophagy, in contrast to STING1-mediated autophagy of the non-dependent ULK1 and BECN1 pathways (127). cGAS has also been shown to bind to dsDNA to form liquid-phase condensates (25). Interestingly, liquid-like condensates can recruit autophagy-related molecules like ATG, LC3, as well as P62 to form cytosomes and participate in the mTOR-mediated autophagic pathway to facilitate cargo degradation (128, 129).

In the immune system, cGAS may be a versatile sensor. Triplet motif containing 14 (TRIM14), a mitochondrial articulator that promotes innate immune signaling, is involved in various tumorigenesis processes. Through the PRYSPRY domain and the C terminus of cGAS, TRIM14 and cGAS interact (130). Researchers demonstrated that TRIM14 inhibits autophagic degradation of cGAS by preventing its entry into the autophagosome, which promotes immune responses (130).

## 9 STING proteins and autophagy

In the drosophila model, previous research revealed that inflammation-induced STING-dependent autophagy limits Zika virus infection (131). In further experiments, it was found that STING may evolve to destroy intracellular pathogens, suggesting that cGAS/STING induces autophagy in an ancient and highly conserved way (132). Nuclear warhead protease B has been found to mediate genomic DNA damage and cell membrane DNA release, activating STING-dependent autophagy and leading to ferrotoxic death in human pancreatic cancer cells (133). This implies that STING-mediated autophagy is potentially promising for the treatment of cancer.

When STING binds to cGAMP, it changes conformation. As the oligomerized STING migrates from the ER to the Golgi apparatus, it passes through the ER-Golgi intermediate compartment (ERGIC). In ERGIC, STING plays an essential role in the induction of autophagy. The STING translocation requires both the COP-II complex and ARF GTPases. The STING-containing ERGIC is capable of lipidating LC3 membranes and thereby triggering the formation of autophagosomes (134). In STING-induced autophagy, the transport of STING from ERGIC to Golgi is unknown. After sensing c-di-AMP, STING disrupts ER homeostasis, leading to the stress of the ER, mTOR inactivation, and ER phagocytosis to coordinate autophagy, thus rescuing dead cells. A recent study has demonstrated that activated STING can undergo intercellular transfer and stimulate RAB22A-mediated non-canonical autophagy derived from the ER, thereby propagating antitumor immunity (135).

Additionally, STING activated the unfolded protein response (UPR) (136). ER stress is induced by unfolded or misfolded proteins, which trigger the UPR to relieve it and restore ER homeostasis. The UPR signaling network activates transcription factor 6, PKR-like ER kinase (PERK), and Inositol-Requiring Protein-1 (137). UPR activation may affect autophagy (138). A lack of PERK has been implicated in converting MDSCs into antitumor CD8^+^ T cells and myeloid immune cells, leading to STING-dependent production of type I IFN and antitumor immunity (115).

By separating ULK1 from AMP-activated proteins, cGAMP generated by cGAS promotes autophagy independent of STING. Upon activation of ULK1, STING is phosphorylated at serine 366, which is then degraded by autophagy and inhibits IRF3 activity (139). In this regard, it is essential to note that, although cGAMP stimulates STING function, it is followed by negative feedback that inhibits the expression of pro-inflammatory molecules, emphasizing the complexity of STING trafficking.

Autophagy proteins have alternative functions, such as LAP, which is involved in phagosome maturation and subsequent signaling mechanisms. Through its direct interaction with LC3, STING mediates autophagy through its classical LIRs. However, STING does not require TBK1 or IRF3 for autophagy to be induced (140). Similarly, autophagy proteins of myeloid cells in the TME are involved in the immunosuppression of T lymphocytes by affecting LAP-induced oncogene expression and triggering the STING-mediated TAM type I IFN response (116).

There is a potential connection between DNA sensing and autophagy: cytosolic DNA inhibits STING-dependent delivery of microbes to autophagosomes that destroy intracellular pathogens (141). The ATG5-dependent autophagy machinery in the ER, which is a key membrane source for autophagosome formation, may regulate innate immune signaling through STING (140). Cytosolic DNA accumulates in cells depleted of ATG5 and ATG7, induced by the expression of STING, STAT1, and ISG15. Activation of the STAT1-ISG15 axis leads to cell migration, invasion, and proliferation, suggesting that inhibition of autophagy can promote tumor-associated phenotypes by activating STING (142). Atg9a is the only multitransmembrane protein identified as an ATG protein in mammals (143) that delivers membranes to the trans-Golgi network (TGN) to form autophagosomes between the plasma membrane and the TGN (144, 145). After dsDNA stimulation, STING colocalizes with the autophagy-associated protein Atg9a and the microtubule-associated protein LC3. When Atg9a is disrupted, the assembly of STING and TBK1 dsDNA is promoted, leading to aberrant activation of innate immunity (146, 147). Interestingly, STING can activate autophagy without Beclin1, Ulk1, or Atg9a (140). A lack of Atg9a led to enhanced STING signaling, suggesting that Atg9a is independent of autophagy in the regulation of STING signaling [118]. Furthermore, activated STING has been reported to recruit ATG16L1 to lipidated LC3 for single membrane perinuclear vesicles through its structural domain WD40, a process that bypasses the requirement for canonical upstream autophagy (148). STING-induced ERGIC or Golgi membrane damage induces the V-ATPase (vacuolar-type H+-ATPase) to lapidate LC3 on the Golgi membrane and participates in non-canonical autophagy (85, 149). These findings suggest that STING can interact with LC3 and participate in noncanonical autophagy.

## 10 Downstream of the cGAS-STING and autophagy

Activating the cGAS-STING pathway can regulate intrinsic cellular programs, such as inducing autophagy in tumor cells (150). Increasing evidence suggests that cargo receptors provide substrates for selective autophagy (151, 152). As a chaperone-like protein, ubiquitously expressed prefoldin like chaperone was vital for suppressing excessive activation of STING1-mediated type I IFN signaling through autophagic degradation of STING1 through sequestosome 1 (153). The Unc-93 homolog B1 attenuates the cGAS-STING signaling pathway by targeting STING for degradation in autophagy lysosomes (154). This provides new insight into the function of STING in innate antiviral immunity, which functions as a checker to prevent hyperactivation.

P62 has been implicated in tumor development as an autophagy selective substrate (155, 156). In cancer cells, increased expression of p62 is associated with defective autophagy, which promotes tumor growth (157). Autophagy can be induced even in the absence of p62 in the presence of ectopic expression of STING (140), indicating that p62 is not necessary for STING-dependent autophagy. The ubiquitination of STING promotes both activation and negative regulation of STING during autophagosome degradation (158). Microtubule-associated protein one LC3 promotes the recruitment of ubiquitinated carriers to the autophagosome membrane through its ubiquitin-associated structural domain. The interaction of LC3-p62 interaction and autophagic degradation is regulated by the structural domain of LIRs (78). By connecting to K63, STING is ubiquitinated and recruited into p62 positive compartments. This results in TBK1 phosphorylating p62 in a manner that depends on IRF3 but not on transcription, thus increasing the affinity of ubiquitin for it. Therefore, p62-deficient cells do not degrade STING, resulting in elevated levels of type I IFN and ISG (159). STING and p62 interact in autophagy and immune regulation, which requires further research.

**Figure 4:**
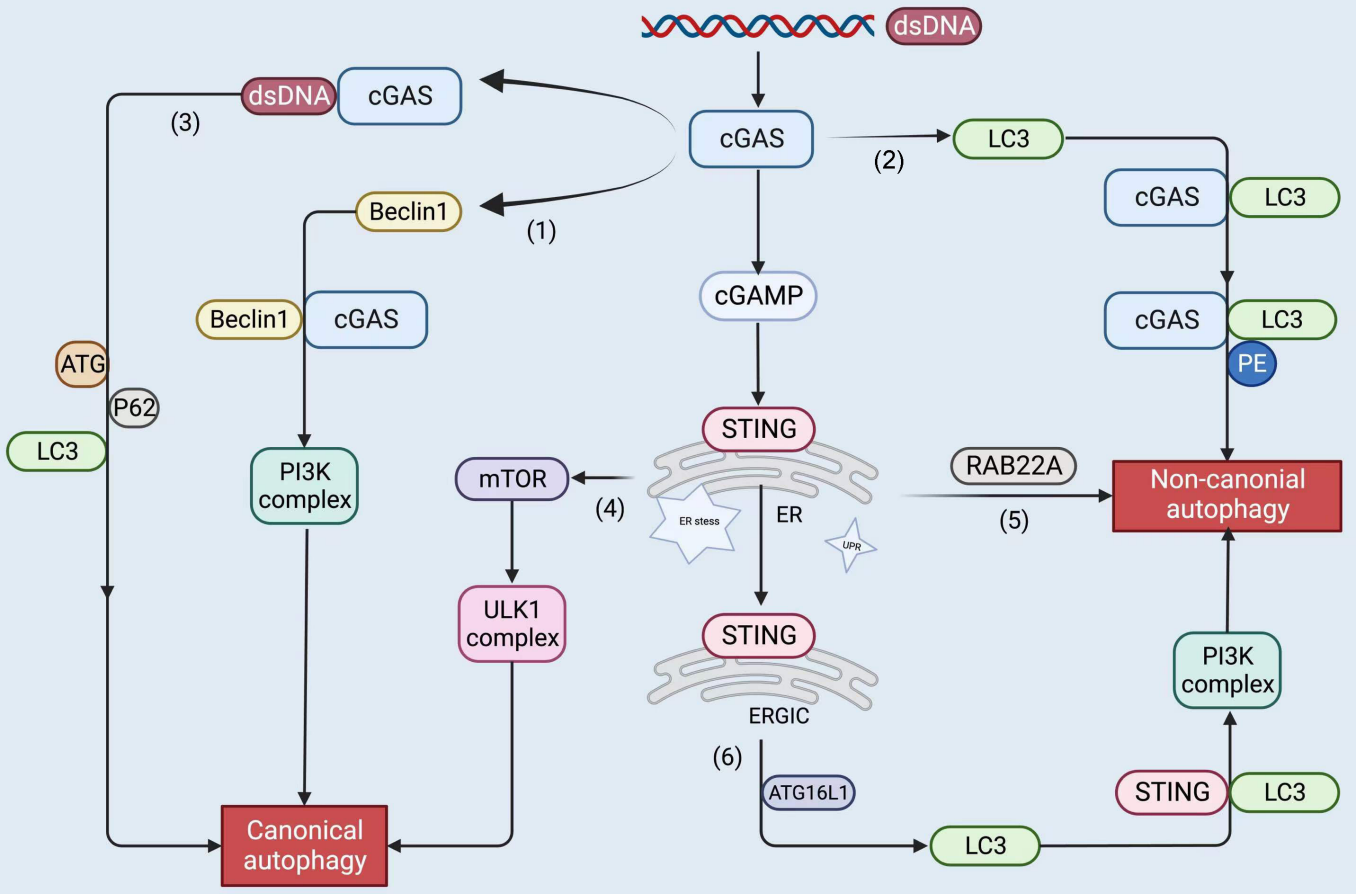
The upstream and downstream of the cGAS-STING pathway, including STING proteins, trigger autophagy by the following roughly divided mechanisms: cGAS binds to dsDNA to form liquid-phase condensates. (1) cGAS interacts with Beclin1 and triggers canonical autophagy; (2) cGAS binds to LC3 to induce non-canonical autophagy; (3) cGAS binds to dsDNA and recruits ATG, LC3, and P62 to participate in canonical autophagy; (4) STING leads to ER stress, mTOR inactivation, and coordinates autophagy; (5) STING stimulates RAB22A-mediated non-canonical autophagy derived from the ER; (6) STING recruits ATG16L1 to lipidated LC3, induces non-canonical autophagy. The figure was created with BioRender (https://biorender.com/).

The mediator of IRF3 activation, a regulator of innate immunity, regulates autophagy flux to promote cell death in breast cancer cells (160). Autophagy may also regulate the stability of IRF3. PSMD14/POH1 deubiquitinase prevents IRF3 autophagy by cleaving its K27-linked polyubiquitin chain in lysine 313 to promote IRF3-mediated type I IFN activation (161). STING also triggers non-canonical autophagy in response to dsDNA, which is crucial for the activation of both IRF3/7 and NF-κB (139). Consequently, selective autophagy-mediated degradation of IRF3 causes immunosuppression by preventing excessive IFN signaling. Nevertheless, IRF3 does not appear to understand the molecular mechanisms that lead to STING degradation. The future of precise immunosuppression may involve activation of the IRF3 pathway, although autophagy may be an important contributor to IRF3-dependent type I IFN signaling (**Figure 4**).

## 11 Discussion and outlook

The cGAS-STING pathway has been identified as a significant immune pathway to recognize cytosolic DNA. It has now made great progress in multiple immune pathways. To support antitumor effects, the host can activate the cGAS-STING pathway, but excessive activation can also contribute to tumor progression. STING activity can be precisely modulated to affect the immune response, including terminating STING-mediated excessive immune activation, which could lead to further investigation. Autophagy exhibits similar dichotomous effects on tumor development. With the advancement of research, autophagy is becoming a more prominent part of tumor immunity. Most of them are focused on the field of canonical autophagy, and non-canonical autophagy remains an area that needs to continue to be explored in depth, which appears to be more comprehensive for better control of mechanistic studies of autophagy in cancer immunity (162).

This review explores the interactions between the upstream and downstream regulators of cGAS-STING and autophagy-related proteins and their relevant effects on cancer immunity. Future research could focus on finding herbal medicine and ingredients that can promote immune cells with antitumor effects. Herbal medicine can be used in combination with chemotherapy or targeted drugs, or immunotherapy represented by PD-1 and PD-L1 inhibitors to have a selective synergistic effect, improving the killing effect of cancer cells, while reducing the side effects of these therapies on healthy ones. In clinical practice, this expectation is consistent with what we have observed. The combination of herbal medicine and various therapies can enhance tumor inhibition more effectively than single drugs (163). Meanwhile, we found that herbal medicine can enhance the cytotoxic effect of chemotherapy on NSCLC by inhibiting cisplatin-induced protective autophagy (164). This way, the application of synergistic treatment of tumors with herbal medicine combined with chemotherapy or targeted drugs, or immunotherapy will be appropriate. This fundamental study can better facilitate the design and development of future antitumor-targeting drugs. Based on the function of cGAS-STING, we will take this pathway as the main means to test the anticancer effect of herbal medicine.

Many interesting questions remain for future investigation and interpretation, although cGAS-STING can trigger both canonical and non-canonical autophagy through multiple pathways. First, in different types of cancer, cGAS-STING inhibits the cell growth cycle through cellular senescence, necrosis, and apoptosis (165–168). What determines cell fate after cGAS-STING mediation? Does the presence of cGAS without transmembrane domains and its localization have an impact on this, including the onset of autophagy? Furthermore, the degree of STING activity and the intrinsic changes in the cancer cells themselves are also taken into account. Second, we need to find other pathways to connect cGAS-STING to autophagy more directly. At present, there is only a preliminary linkage between the two, but there is no more comprehensive systematic evidence to combine them and coordinate a series of downstream pathways to improve tumor immune efficiency in response to various foreign stimuli. Third, it is worthwhile to think about how to more fully elucidate the specific structures and modes of interaction between STING and some of the factors associated with autophagy along with drug trials and applications concerning each of them. Overall, combining cGAS-STING with autophagy can help to deepen the understanding of the intersection of innate and acquired immunity, which provides a new avenue for studying antitumor immunity.

## Author contributions

QL wrote the first version of the manuscript, and YC finalized the manuscript. JL downloaded the references and processed the figures in the manuscript. FZ collected the data. ZZ (corresponding author) conceived and coordinated the study and critically evaluated the data. All authors contributed to the article and approved the submitted version.

## Glossary

**Table:**

PD-1/PD-L1	programmed cell death protein 1/programmed cell death ligand 1
CAR-T cell	chimeric antigen receptor T-cell
DCs	dendritic cells
ILCs	innate lymphocytes
NK	natural killer
PRRs	pattern recognition receptors
PAMPs	pathogen-associated molecular patterns
DAMPs	danger-associated molecular patterns
cGAS	cyclic guanosine monophosphate-adenosine monophosphate synthase
dsDNA	double-stranded DNA
STING	stimulator interferon gene
ER	endoplasmic reticulum
cGAMP	cyclic guanosine monophosphate-adenosine monophosphate
IFN	interferon
APCs	antigen-presenting cells
STAT1	signal transducer and activator of transcription 1
EMT	epithelial-mesenchymal transition
TME	tumor microenvironment
ISG	invariant surface glycoprotein
cGAMP-NP	cGAMP nanoparticles
MDSCs	myeloid derived suppressor cells
IDO	indolamine 2,3-dioxygenase
LKB1	liver kinase B1
NSCLC	non-small cell lung cancer
CMA	chaperone-mediated autophagy
ATG	autophagy-related gene
PI3K	phosphoinositide 3-kinase
PE	phosphatidylethanolamine
PI3KC3	class III PI3K
ROS	reactive oxygen species
FoxO	Forkhead box O
UBD	ubiquitin binding domain
MHC	major histocompatibility complex
PDAC	pancreatic ductal adenocarcinoma
LAP	LC3-associated phagocytosis
TAMs	tumor-associated macrophages
Gal-1	galectin-1
HCC	Hepatocellular carcinoma
LIRs	LC3-interacting regions
TRIM14	triplet motif containing 14
ERGIC	ER–Golgi intermediate compartment
UPR	unfolded protein response
PERK	PKR-like ER kinase
TGN	trans-Golgi network
