# Corrigendum: Crosstalk between cGAS-STING pathway and autophagy in cancer immunity

**DOI:** 10.3389/fimmu.2023.1216456

**Published:** 2023-05-19

**Authors:** Qijun Lu, Yukun Chen, Jianwen Li, Feng Zhu, Zhan Zheng

**Affiliations:** ^1^ Department of Oncology, Longhua Hospital, Shanghai University of Traditional Chinese Medicine, Shanghai, China; ^2^ Cancer Institute, Longhua Hospital, Shanghai University of Traditional Chinese Medicine, Shanghai, China; ^3^ Department of Laboratory Medicine, Huadong Hospital, Fudan University, Shanghai, China

**Keywords:** antitumor, autophagy, cancer, cGAS-STING, immunity

In the published article, there was an error in **Figure 1** as published. The word ‘immunity’ is not capitalized, keeping the format consistent. The corrected **Figure 1** and its caption “**Figure 1**. appear below.

**Figure 1:**
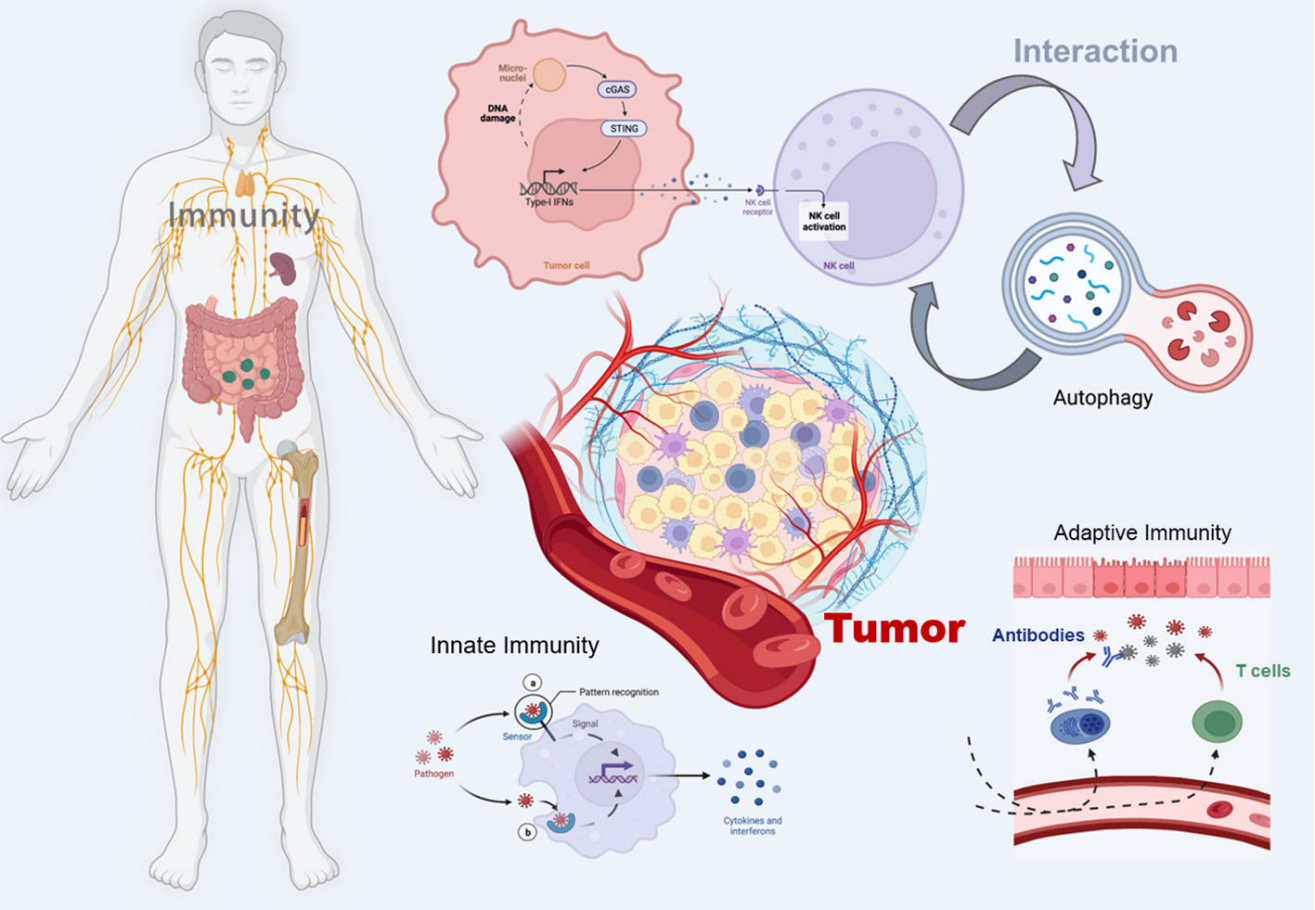
Schematic illustration of the crosstalk between the cGAS-STING pathway and autophagy in cancer immunity. The figure was created with BioRender.

In the published article, there was an error in **Figure 4** as published. The icon (2) in **Figure 4** is missing. The corrected **Figure 4** and its caption “**Figure 4** appear below.

**Figure 4:**
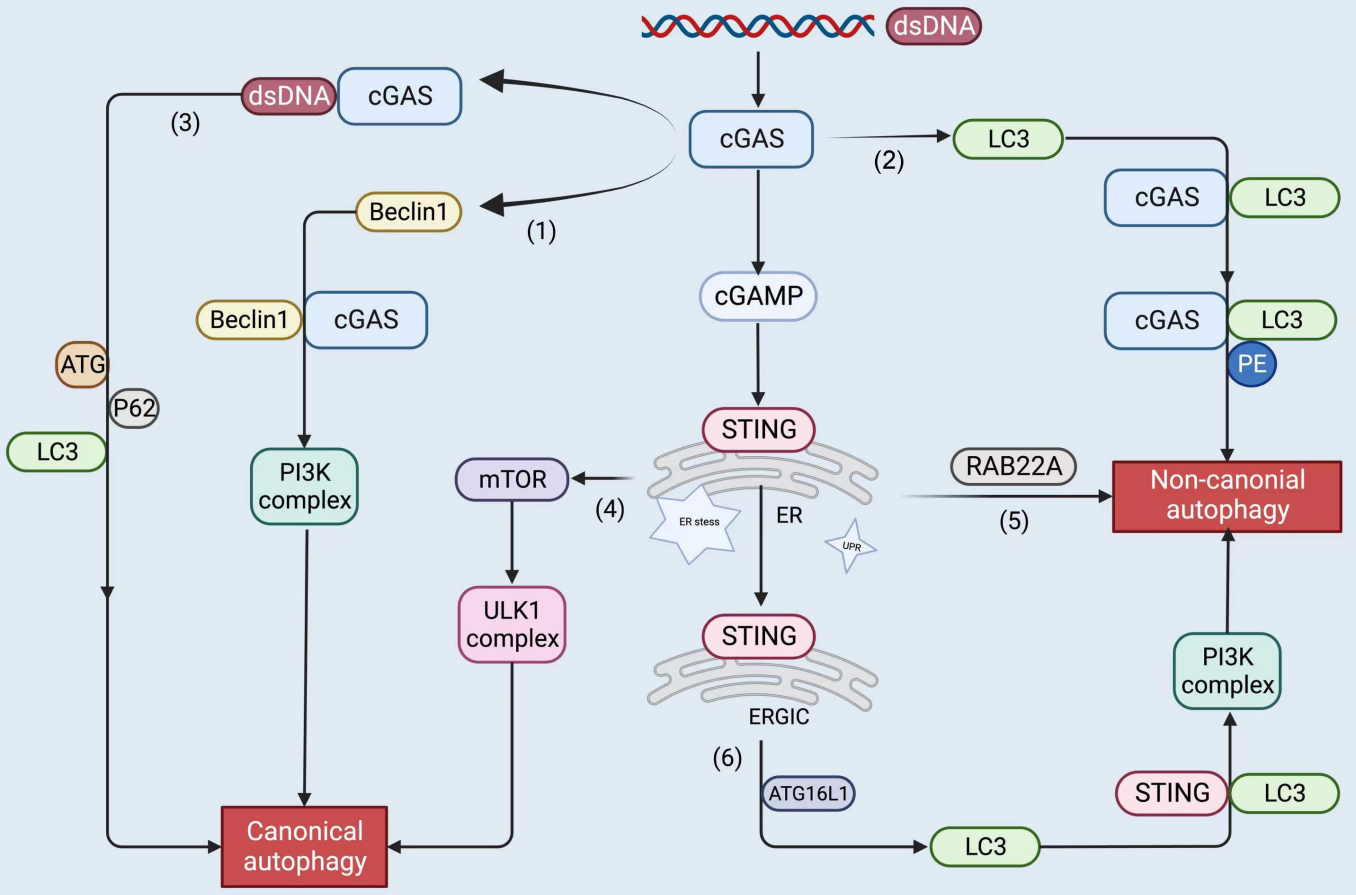
The upstream and downstream of the cGAS-STING pathway, including STING proteins, trigger autophagy by the following roughly divided mechanisms: cGAS binds to dsDNA to form liquid-phase condensates. (1) cGAS interacts with Beclin1 and triggers canonical autophagy; (2) cGAS binds to LC3 to induce non-canonical autophagy; (3) cGAS binds to dsDNA and recruits ATG, LC3, and P62 to participate in canonical autophagy; (4) STING leads to ER stress, mTOR inactivation, and coordinates autophagy; (5) STING stimulates RAB22A-mediated non-canonical autophagy derived from the ER; (6) STING recruits ATG16L1 to lipidated LC3, induces non-canonical autophagy. The figure was created with BioRender.

In the published article, there was an error in the author list, and author Yukun Chen was erroneously denoted as co-first author. The corrected author list appears below.

Qijun Lu^1^, Yukun Chen^2^, Jianwen Li^1^, Feng Zhu^3^ and Zhan Zheng^1*^

The authors apologize for these errors and state that this does not change the scientific conclusions of the article in any way. The original article has been updated.

